# Stimulus-induced EEG-patterns and outcome after cardiac arrest

**DOI:** 10.1016/j.cnp.2021.07.001

**Published:** 2021-07-21

**Authors:** N.Jaffer Broman, S. Backman, E. Westhall

**Affiliations:** Lund University, Skane University Hospital, Department of Clinical Sciences, Clinical Neurophysiology, Lund, Sweden

**Keywords:** ACNS, American Clinical Neurophysiology Society, CPC, Cerebral Performance Category, IQR, interquartile range, NSE, neuron-specific enolase, SIRPIDs, stimulus-induced rhythmic, periodic or ictal discharges, SI-PD, stimulus-induced periodic discharges, SI-RDA, stimulus-induced rhythmic delta activity, SI-SW, stimulus-induced spike-/polyspike-/sharp-and-waves, SI-Seizures, stimulus-induced unequivocal seizures, TTM, targeted temperature management, Cardiac arrest, EEG, SIRPIDs, Prognosis, Coma

## Abstract

**Objective:**

EEG is commonly used to predict prognosis in post anoxic coma. We investigated if stimulus-induced rhythmic, periodic or ictal discharges (SIRPIDs) add prognostic information after cardiac arrest.

**Methods:**

In the multicenter Targeted Temperature Management trial, routine-EEGs were prospectively recorded after rewarming (≥36 h). Presence and subtype of SIRPIDs and main EEG-pattern (benign, malignant, highly malignant) were retrospectively reported according to a standardised classification. Patients were followed up after 180 days. Poor outcome was defined as severe neurological disability or death (Cerebral Performance Category 3–5).

**Results:**

Of 142 patients, 71% had poor outcome and 14% had SIRPIDs. There was no significant difference in outcome between patients with and without SIRPIDs, even when subgrouped according to underlying main EEG-pattern. Comparing subtypes of SIRPIDs, 82% of patients with stimulus-induced periodic discharges had poor outcome compared to 44% of patients with stimulus-induced rhythmic delta activity, but the difference was not significant.

**Conclusions:**

In EEGs performed ≥36 h after cardiac arrest, SIRPIDs cannot be used to reliably predict poor outcome. Whether certain subtypes of SIRPIDs indicate worse prognosis needs further investigation.

**Significance:**

Categorising the main EEG-pattern has important prognostic implications, but assessment of late appearing SIRPIDs does not seem to add prognostic information.

## Highlights

• Presence of SIRPIDs on a late routine-EEG adds no reliable prognostic information.
• SIRPIDs was rare among patients with a highly malignant EEG.
• Whether specific subtypes of SIRPIDs have prognostic implications needs further investigation.

## 1 Introduction

Post cardiac arrest patients who are still comatose after rewarming become subject for neurological prognostication to assess the degree of hypoxic-ischemic brain injury (Nolan et al., 2015). The prognostic tools include clinical neurological examination, biomarkers in serum, neuroradiology, somatosensory evoked potentials (SSEP) and electroencephalography (EEG). One biomarker commonly used for prognostication is neuron-specific enolase (NSE) (Stammet et al., 2015), which is an enzyme found in neurons and is released during brain injury (Rundgren et al., 2009). Routine EEG is the most commonly used prognostic tool (Friberg et al., 2015), but lack of standardised interpretation has limited the level of evidence. Based on standardised terminology from the American Clinical Neurophysiology Society (ACNS) (Hirsch et al., 2013), benign, malignant and highly malignant EEG-patterns have been proposed for post cardiac arrest prognostication (Westhall et al., 2014). In the prospective Targeted Temperature Management trial (TTM trial), the highly malignant patterns were found to be robust predictors of poor outcome and the benign patterns predictive of good outcome (Backman et al., 2018, Westhall et al., 2016).

Stimulus-induced rhythmic, periodic or ictal discharges (SIRPIDs) were first described in 2004 and were hypothesised to reflect a dysregulation of projections to an injured cortex (Hirsch et al., 2004). SIRPIDs are present in 10–34% of critically ill patients, but the clinical implications are unclear (Johnson et al., 2018). One study reported a 13% rate of SIRPIDs after cardiac arrest and found an association to poor neurological outcome, especially when SIRPIDs occurred early during ongoing hypothermia (Alvarez et al., 2013). Another small study found 15% SIRPIDs 2–3 days after cardiac arrest and reported an association with poor outcome but with a high false positive rate (Benarous et al., 2019).

In the ACNS-terminology, the stimulus-induced (SI) patterns are subcategorised into rhythmic delta activity (SI-RDA), periodic discharges (SI-PD), spike-/polyspike-/sharp-and-wave (SI-SW) or unequivocal seizure activity (SI-Seizures) (Hirsch et al., 2013). Whether these subtypes have different prognostic implications after CA has not been previously investigated.

In the multicenter TTM-trial, two target temperatures, 33 °C and 36 °C, were compared after cardiac arrest and no differences were found regarding outcome or levels of NSE (Nielsen et al., 2013). A routine-EEG was included in the study protocol. Using this EEG-data, the aim of the present study is to investigate the association between SIRPIDs and poor outcome in comatose survivors of cardiac arrest addressed by the following predefined hypotheses:

• Patients with SIRPIDs will have a higher rate of poor outcome as compared to patients without SIRPIDs (main hypothesis).
• An association between SIRPIDs and poor outcome will remain, even when subcategorised according to the main EEG-pattern (benign, malignant, highly malignant).
• The level of a serum biomarker of brain injury (NSE) will be higher among patients with SIRPIDs compared to patients without SIRPIDs.

## 2 Methods

### 2.1 Patients

Between 2010 and 2013, 939 adult patients with out-of-hospital cardiac arrest of presumed cardiac cause at 36 sites in Europe and Australia were included in the TTM-trial (Nielsen et al., 2013). The detailed protocol has been published (Nielsen et al., 2012). The trial was approved by the ethics committees in participating countries.

After randomisation, patients were cooled to their assigned target temperature. Patients were rewarmed and normothermic at a time-point corresponding to 36 h after cardiac arrest. Sedation was tapered, if not needed for intensive care reasons. Neuroprognostication 72 h after rewarming was based on clinical neurological evaluation, along with SSEP and EEG. A decision on withdrawal or continuation of life-supporting therapy was made. Criteria for withdrawal of life-supporting therapy were pre-specified (Nielsen et al., 2012).

### 2.2 EEG

Routine EEGs were performed according to the trial protocol in patients who remained unconscious after rewarming. Consecutive patients with an EEG recorded 36 h to 10 days after cardiac arrest were included in the central EEG database of the TTM-trial. The trial protocol recommended at least 16 electrodes, reference and ground placed according to the international SI-system and a duration of the recording of 20 min. Recordings with less than 12 electrodes or a duration less than 10 min were excluded. Parts of this EEG-data has previously been published (Backman et al., 2018, Westhall et al., 2016), but the prognostic value of SIRPIDs has not been reported. The EEG-reviews were performed blinded to all clinical data and to outcome using a structured electronic case report form based on the terminology proposed by ACNS (Hirsch et al., 2013).

In the present study, all EEGs with reactivity testing were included if testing was performed according to the protocol, with at least two repetitions for both auditory and painful stimuli. The two recommended types of auditory stimuli according to protocol were hand clapping and saying the patient’s name. The painful stimuli were recommended to include central pain stimulation. EEGs were excluded if notations regarding reactivity testing were lacking due to technical reasons in the exported files. The presence and type of SIRPIDs were reported. Interpreters marked the presence of SIRPIDs as either yes, no or unclear and subclassified into SI-RDA, SI-PD, SI-SW or SI-Seizures.

The main patterns of the EEGs have been classified as either benign, malignant or highly malignant according to pre-specified criteria (Westhall et al., 2014) based on the proposed ACNS terminology. Highly malignant patterns were defined as a suppressed background with or without periodic discharges or burst suppression, with or without discharges. Malignant patterns were defined as discontinuous background, low-voltage background, reversed anterio-posterior gradient, abundant periodic or rhythmic discharges or seizures. Background reactivity was not included in the definition of malignant patterns. A benign pattern was defined as a continuous background with normal voltage and absence of any malignant features stated above.

### 2.3 Follow-up

Neurological function was graded during the hospital stay and at follow-up 180 days after cardiac arrest according to the Cerebral Performance Category Scale (CPC scale, 1 = no/minor disability, 2 = moderate disability, 3 = severe disability, 4 = coma/vegetative state, 5 = death) (Jennett and Bond, 1975). Poor outcome was defined as a best CPC value of 3–5.

### 2.4 Neuron-specific enolase

In the TTM-trial, serum levels of NSE was analysed using a COBAS e601 line with an Electro-Chemi-Luminescent-Immuno-Assay kit (Roche diagnostics). Details regarding NSE sampling and analysis in the TTM-trial have been published (Stammet et al., 2015). The NSE analysis was performed after the trial and NSE was not included in the recommendations for decisions regarding level of care. The maximum value measured at 48 and 72 h after cardiac arrest was used in the present study.

### 2.5 Statistics

SPSS 25.0 was used. Jeffrey’s method was used to calculate the confidence intervals for percentages. Fisher’s exact test was used to calculate p-values for all binary outcomes. For analyses on continuous data, Mann-Whitney *U* test was used. A p-value of <0.05 was considered significant. Data on NSE was presented using median values and interquartile range (IQR), as well as range where the number of data points was small. The odds ratio for outcome was calculated using logistic regression.

## 3 Results

### 3.1 Patients

**Fig. 1:**
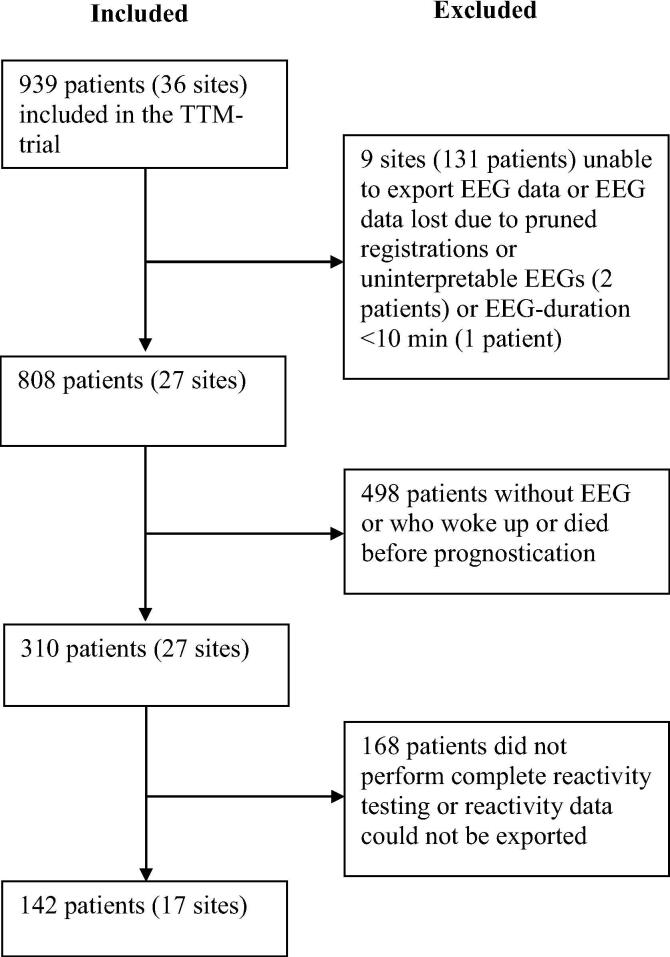
Flow chart for inclusion in the study.

142 patients from 17 sites who had EEGs with reactivity testing available were included in the present study. The inclusion flow chart is presented in Fig. 1. Baseline characteristics are presented in Table 1. The median time from cardiac arrest to EEG was 73 h (IQR 53–99). The rate of poor outcome was 71%.

**Table 1:** A comparison of descriptive characteristics between patients with and without SIRPIDs.

	Patients with SIRPIDs (n = 20)	Patients without SIRPIDs (n = 122)	P-value
Age, mean years ± standard deviation	66 ± 12	66 ± 11	p = 0.951
Sex, no of males (%)	15 (75%)	100 (82%)	p = 0.538
Comorbidities before randomisation, no (%)			
Ischemic heart disease	4 (20%)	37 (30%)	p = 0.432
Arterial hypertension	6 (30%)	65 (54%)	p = 0.089
Previous stroke or transient ischemic attack	2 (10%)	13 (10%)	p = 1.000
Diabetes mellitus	1 (5%)	20 (17%) Missing: 3	p = 0.309
Epilepsy	0 (0 %)	0 (0 %)	Not applicable
Variables related to cardiac arrest			
Bystander-witnessed cardiac arrest, no (%)	18 (90%)	113 (93%)	p = 0.654
Shockable first rhythm, no (%)	18 (90%)	83 (68%)	p = 0.061
Time to return of spontaneous circulation, median minutes (IQR)	22 (20–46)	30 (22–45)	p = 0.244
Randomised to 36 °C, no (%)	7 (35%)	66 (54%)	p = 0.148
Time to ICU discharge/death after cardiac arrest, median hours (IQR)	192 (148–327)	147 (115–230)	p = 0.053
Time to EEG after cardiac arrest, median hours (IQR)	89 (67–124)	72 (52–96)	p = 0.010

SIRPIDS = stimulus-induced rhythmic, periodic or ictal discharges. IQR = interquartile range. ICU = intensive care unit.

### 3.2 SIRPIDs compared to non-SIRPIDs

Of 142 patients, 20 patients presented SIRPIDs on their EEG-recording, resulting in a prevalence of 14% (95% CI 9–21). There were no significant differences in outcome or levels of NSE when comparing patients with and without SIRPIDs (Table 2). A highly malignant EEG was rare among patients with SIRPIDs compared to the group of patients without SIRPIDs (p = 0.002).

**Table 2:** A comparison between patients with and without SIRPIDs in regard to EEG-characteristics, maximum NSE-levels and rate of poor outcome.

	Patients with SIRPIDs (n = 20)	Patients without SIRPIDs (n = 122)	P-value	OR (95% CI)
Highly malignant EEG, n (%)	1 (5%)	48 (39%)	p = 0.002	
Malignant EEG, n (%)	9 (45%)	40 (33%)	p = 0.316	
Benign EEG, n (%)	10 (50%)	34 (28%)	p = 0.067	
Unreactive background, n (%)	12 (60%)	79 (65%)	p = 0.802	
Ongoing sedation during EEG	4 (20%)	42 (36%) Missing: 5	p = 0.206	
NSE, median ng/ml (IQR)	35 (10–91) Missing: 7	47 (16–151) Missing: 20	p = 0.160	
Poor outcome (CPC 3–5), n (%)	13 (65%)	88 (72%)	p = 0.596	0.7 (95% CI 0.3 –2.0)

SIRPIDs = stimulus-induced rhythmic, periodic or ictal discharges. NSE = neuron specific enolase. CPC = cerebral performance category. IQR = interquartile range. OR = odds ratio. CI = confidence interval.

### 3.3 Comparisons of SIRPID-subgroups

**Fig. 2:**
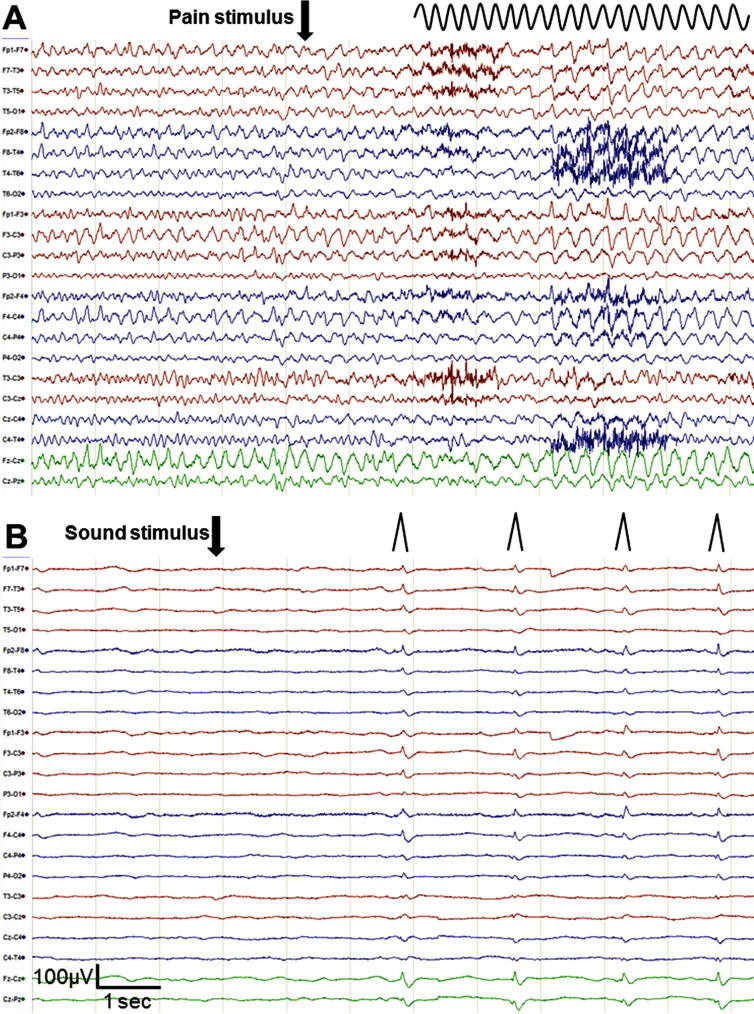
A, Stimulus-induced rhythmic delta activity (SI-RDA) (sharply contoured) superimposed on a benign main EEG-pattern (continuous normal-voltage background > 20 µV). B, Stimulus-induced periodic discharges (SI-PD) superimposed on a malignant main EEG-pattern (low-voltage background 10–20 µV).

Of 20 patients with SIRPIDs, 9 (45%) were classified as SI-RDA and 11 (55%) as SI-PD (see Fig. 2 for EEG-examples). All SIRPIDs had a generalized localisation. None of the SIRPID-patterns were classified as SI-SW or SI-Seizures. Comparisons of SI-RDA and SI-PD are presented in Table 3. The rate of poor outcome was 82% among patients presenting with SI-PD compared to 44% in patients with SI-RDA, but this difference did not reach statistical significance (p = 0.160). There were no significant differences in NSE values comparing SI-PD with SI-RDA.

**Table 3:** A comparison between patients with SI-RDA and SI-PD in regard to EEG-characteristics, maximum NSE-levels and rate of poor outcome.

	SI-RDA (n = 9)	SI-PD (n = 11)	P-value	OR (95% CI)
Highly malignant EEG, n (%)	0	1 (9%)	p = 1.000	
Malignant EEG, n (%)	3 (33%)	6 (55%)	p = 0.406	
Benign EEG, n (%)	6 (67%)	4 (36%)	p = 0.370	
Unreactive background, n (%)	4 (44%)	8 (73%)	p = 0.362	
NSE, median ng/ml (IQR)	28 (10 – 51) Missing: 3	35 (10 – 110) Missing: 4	p = 0.731	
Poor outcome (CPC 3–5), n (%)	4 (44%)	9 (82%)	p = 0.160	5.6 (0.7–42.4)

SIRPIDs = stimulus-induced rhythmic, periodic or ictal discharges. SI-RDA = stimulus-induced rhythmic delta activity. SI-PD = stimulus-induced periodic discharges. NSE = neuron specific enolase. CPC = cerebral performance category. IQR = interquartile range. OR = odds ratio. CI = confidence interval.

### 3.4 SIRPIDs categorised according to main EEG-pattern

Subcategorising SIRPIDs according to which main EEG-pattern they were superimposed on and type of SIRPID (SI-PD or SI-RDA) are presented in Table 4 (outcome data) and Supplementary Table e-1 (NSE data). None of the comparisons in these tables reached significance. The majority of patients with a benign EEG, with or without SIRPIDs had a good outcome. Among patients with a malignant EEG, there were no significant differences in poor outcome when comparing patients with or without SIRPIDs. Comparing subtypes of SIRPIDs among patients with a malignant EEG, poor outcome was seen in 100% (6/6) of patients with SI-PD and in 67% (2/3) of the patients with SI-RDA, but this difference was not statistically significant. There were no significant differences in levels of NSE comparing these groups.

**Table 4:** A comparison of outcome between patients with and without SIRPIDs, and between SIRPID-subgroups, categorised according to underlying main EEG-pattern.

	Poor outcome (CPC 3–5), n (%)	P-value
**Benign EEG (n = 44)**	10 (23%)	
Benign EEG without SIRPIDs (n = 34)	6 (18%)	p = 0.199
Benign EEG with SIRPIDs (n = 10)	4 (40%)	
*Benign EEG with SI-RDA (n = 6)*	2 (33%)	p = 1.000
*Benign EEG with SI-PD (n = 4)*	2 (50%)	
**Malignant EEG (n = 49)**	43 (88%)	
Malignant EEG without SIRPIDs (n = 40)	35 (88%)	p = 1.000
Malignant EEG with SIRPIDs (n = 9)	8 (89%)	
*Malignant EEG with SI-RDA (n = 3)*	2 (67%)	p = 0.333
*Malignant EEG with SI-PD (n = 6)*	6 (100%)	

SIRPIDs = stimulus-induced rhythmic, periodic or ictal discharges. SI-RDA = stimulus-induced rhythmic delta activity. SI-PD = stimulus-induced periodic discharges. CPC = cerebral performance category.

## 4 Discussion

This study on comatose patients after cardiac arrest confirms that SIRPIDs is relatively common (14%). The patients with SIRPIDs in the studied time-window (36 h to 10 days after cardiac arrest) did not have a higher rate of poor outcome compared to patients without SIRPIDs. Thus, the main hypothesis of the study was discarded. The subtype SI-PD had a higher rate of poor outcome compared to the subtype SI-RDA, but the difference was not significant in this study with limited power in subgroups and no conclusions can be drawn. No significant differences in outcome were found when comparing subgroups of patients with a benign EEG with or without SIRPIDs, nor patients with a malignant EEG with or without SIRPIDs.

The present study goes against the previously published studies on SIRPIDs after cardiac arrest (Alvarez et al., 2013; Benarous et al., 2019), which found an association between SIRPIDs and poor outcome. However, in the study by Alvarez *et al*, EEGs were performed both during ongoing hypothermia and later during normothermia. The authors emphasized that SIRPIDs were particularly associated with poor outcome during the early time-window after cardiac arrest during ongoing hypothermia and sedation. Further, they reported only few patients with SI-RDA, which seemed less malignant in our study. They had some patients with stimulus-induced ictal patterns (SI-seizures), which may be prognostically worse considering that spontaneous unequivocal seizures are strongly associated with poor outcome (Backman et al., 2017). A study on patients with postanoxic epileptiform EEGs, including status epilepticus, reported that if SIRPIDs appeared late, beyond 36 h after cardiac arrest, it was a favourable sign, but subgroups of SIRPIDs were not presented (Barbella et al., 2020).

In the present study, the rate of SIRPIDs was 14%, which is similar to previous studies after cardiac arrest (Alvarez et al., 2013; Benarous et al., 2019), and in line with the prevalence among critically ill patients (Alsherbini et al., 2017, Braksick et al., 2016, Van Straten et al., 2014). SIRPIDs, however, seem to be transient in nature. In the study by Alvarez *et al*, no patients had SIRPIDs during both hypothermia and normothermia (Alvarez et al., 2013). This apparent transience is further emphasised by its reported higher prevalence in prolonged continuous EEG-monitoring (Johnson et al., 2018). Considering this, it is possible that we would have found a higher prevalence with the use of repeated or continuous EEG-monitoring. Additionally, there exists interrater variability in identifying SIRPIDs. Interrater agreement for SIRPIDs in the present cohort was only slight, kappa value 0.19 (Westhall et al., 2015). Another study reported moderate interrater agreement (Alsherbini et al., 2017). The low interrater agreement may limit the use of SIRPIDs for prognostication.

The only subcategories of SIRPIDs found in this study were SI-PD and SI-RDA. Reports on most common subtype vary between ictal-appearing (SI-seizures) (Hirsch et al., 2004), SI-RDA (Alsherbini et al., 2017) and SI-PD (Alvarez et al., 2013, Braksick et al., 2016). Further, there is still variability among the definitions of subtypes used in studies despite the suggested ACNS-terminology, rendering comparisons difficult.

Our comparisons between the subtypes SI-PD and SI-RDA could not exclude that SI-PD might be predictive of a worse outcome, since we observed a large effect (odds ratio 5.6) but with wide confidence intervals and the difference failed to reach significance (p = 0.160), possibly due to few patients in these subgroups. Further, there was no significant difference in NSE-levels comparing SI-PD and SI-RDA. Patients with SI-RDA had a lower rate of poor outcome than the overall rate for the cohort, suggesting that SI-RDA may even be associated with a better prognosis after cardiac arrest, resembling spontaneously appearing RDA which is associated with good outcome (Soholm et al., 2014). However, there were few patients in this cohort with SI-RDA, and thus no conclusions can be drawn.

It is interesting that SIRPIDs was very rare among patients with a highly malignant EEG. Alvarez *et al* found only few patients with SIRPIDs among those with the highest NSE-values. They hypothesised that if the brain was too injured, it may be unable to produce SIRPIDs (Alvarez et al., 2013). Our finding that only one patient with SIRPIDs had a highly malignant EEG could support this theory. Due to the fact that a highly malignant EEG was so uncommon in patients with SIRPIDs, it was excluded from further subanalyses. If presence of SIRPIDs indicate lack of extensive brain injury this might be useful when identifying patients with postanoxic status epilepticus that have a potential for good outcome. Barbella *et al* suggested that late appearing SIRPIDs might be a favourable sign in patients with an epileptiform EEG (Barbella et al., 2020).

The majority of patients with a benign EEG and SIRPIDs survived with a good outcome. A malignant EEG has lower predictive ability compared to highly malignant and benign patterns, and should be considered a prognostically intermediate pattern (Backman et al., 2018, Westhall et al., 2016). Therefore, combining it with SIRPIDs is interesting from a clinical standpoint. However, for patients with a malignant EEG, SIRPIDs did not seem to add any prognostic information. One of the patients with both a malignant EEG and SIRPIDs survived with good outcome, indicating that the combination of a malignant EEG and SIRPIDs cannot be used to reliably predict outcome. Although there were no survivors with good outcome in the group with malignant EEG and SI-PD, only six patients presented with this combination and no statistical significance was found. A suggestion for a future study would be to investigate the prognostic ability of malignant EEG with SI-PD in a larger cohort.

This study has several limitations. EEG was missing for many patients due to technical difficulties with the export of the EEGs which may limit the generalizability of the results and reduces the sample size. For the same reasons, no multivariate analysis was conducted. Another limitation is that EEGs were only performed after rewarming since SIRPIDs are transient and the predictive value of SIRPIDs may be time-dependent.

### 4.1 Conclusions

SIRPIDs are relatively common in comatose patients after cardiac arrest. When SIRPIDs are present on EEGs performed during normothermic conditions, they do not appear to add any prognostic information compared to assessing only the main EEG-pattern (highly malignant, malignant, benign). However, whether the subtype SI-PD is associated with a worse outcome compared to the subtype SI-RDA needs to be addressed in a future study on a larger patient cohort.

## Funding

The Swedish Heart and Lung Association; the Skåne University Hospital Foundations; the Gyllenstierna-Krapperup Foundation; the 10.13039/501100006686Segerfalk foundation; the Swedish National Health System (ALF); the County Council of Skåne; the 10.13039/501100007687Swedish Society of Medicine; the Koch Foundation, Sweden, The Swedish Heart-Lung Foundation, 10.13039/501100002706AFA Insurance, The Swedish Research Council and 10.13039/501100017237Hans-Gabriel and Alice Trolle-Wachtmeister Foundation; all in Sweden. The Tryg Foundation; Denmark. EU programme Interreg IV A. The funding sources had no involvement in the collection, analysis and interpretation of data, in the writing of the manuscript or in the decision to submit the manuscript.
