# Supplementary material for: Stimulus-induced EEG-patterns and outcome after cardiac arrest
Source: Clin Neurophysiol Pract. 2021 Jul 21;6:219–24. doi: 10.1016/j.cnp.2021.07.001 (PMC8350459; doi:10.1016/j.cnp.2021.07.001)
Supplement: Supplementary data 1 [file mmc1.docx]

**Supplementary table e-1.**

NSE-levels for SIRPIDs and SIRPID subgroups categorised according to main EEG-pattern

|  | **NSE, median ng/ml** | **IQR** | **Range** | **P-value** |
| --- | --- | --- | --- | --- |
| **Benign EEG** **(n=32)** | 13 | 9 – 20 | 4 – 119 |  |
| Benign EEG  without SIRPIDs (n=26) | 13 | 9 – 19 | 4 – 119 | p=0.683 |
| Benign EEG  with SIRPIDs (n=6) | 15 | 9 – 37 | 8 – 42 |  |
| *Benign EEG*  *with SI-RDA* *(n=3)* | 10 | n/a | 9 – 42 | p=1.000 |
| *Benign EEG*  *with SI-PD* *(n=3)* | 20 | n/a | 8 – 35 |  |
|  |  |  |  |  |
| **Malignant EEG** **(n=41)** | 48 | 21 – 119 | 3 – 274 |  |
| Malignant EEG  without SIRPIDs (n=34) | 40 | 20 – 128 | 3 – 274 | p=0.986 |
| Malignant EEG  with SIRPIDs (n=7) | 78 | 21 – 110 | 10 – 123 |  |
| *Malignant EEG*  *with SI-RDA (n=3)* | 36 | n/a | 21 – 78 | p=0.400 |
| *Malignant EEG*  *with SI-PD* *(n=4)* | 108 | n/a | 10 – 123 |  |

A comparison of NSE-levels between patients with and without SIRPIDs, and between SIRPID-subgroups, categorised according to underlying main EEG-pattern. Only patients with available NSE data is presented. IQR is not presented in groups where number of patients were too small. SIRPIDs = stimulus-induced rhythmic, periodic or ictal discharges. SI-RDA = stimulus-induced rhythmic delta activity. SI-PD = stimulus-induced periodic discharges. NSE = neuron-specific enolase. IQR = interquartile range. n/a = not applicable.
